# CO_2_-Rich Industrial Waste Gas as a Storage-Enhanced Gas: Experimental Study on Changes in Pore Structure and Methane Adsorption in Coal and Shale

**DOI:** 10.3390/molecules30122578

**Published:** 2025-06-13

**Authors:** Hanxin Jiu, Dexiang Li, Gongming Xin, Yufan Zhang, Huaxue Yan, Tuo Zhou

**Affiliations:** 1 School of Nuclear Science, Energy and Power Engineering, Shandong University, Jinan 250061, China; 202314510@mail.sdu.edu.cn (H.J.);; 2 CNPC Engineering Technology R & D Company Limited, Beijing 102206, China

**Keywords:** CO_2_-rich industrial waste gas, pore volume, specific surface area, adsorption capacity, connectivity, coal-bearing rock series

## Abstract

A technology that directly injects CO_2_-rich industrial waste gas (CO_2_-rich IWG) into underground spaces for unconventional natural gas extraction and waste gas storage has received increasing attention. The pore characteristics of coal and shale in a coal-bearing rock series before and after CO_2_-rich IWG treatment are closely related to gas recovery and storage. In this study, three coals ranging from low to high rank and one shale sample were collected. The samples were treated with CO_2_-rich IWG using a high-precision geochemical reactor. The changes in the pore volume (PV), specific surface area (SSA), and pore size distribution of micropores, mesopores, and macropores were analyzed. The correlations between the Langmuir volume and the PV and SSA of the micropores and mesopores were analyzed. It was confirmed that for micropores, SSA was the dominant factor influencing adsorption capacity. The effectively interconnected pore volume was calculated using macropores to characterize changes in the sample’s connectivity. It was found that the PV and SSA of the micropores in the coal samples increased with increasing coal rank. The CO_2_-rich IWG treatment increased the PV and SSA of the micropores in all of the samples. In addition, for mesopores and macropores, the treatment reduced the SSA in the coal samples but enhanced it in the shale. The results of this study improve the understanding of the mechanisms of the CO_2_-rich IWG treatment method and emphasize its potential in waste gas storage and natural gas extraction.

## 1. Introduction

With rapid global industrialization and urbanization, environmental issues are becoming a major constraint on sustainable economic development. It is thus a priority to control the emission of greenhouse gases, such as CO_2_, while transitioning to an ecological, civilization-oriented development strategy [1]. CO_2_-enhanced gas recovery (CO_2_-EGR) is a technique for extracting methane gas from coal beds or shales by injecting CO_2_ gas. It can store CO_2_ underground and contribute to carbon capture and storage (CCS) initiatives [2,3,4]. According to an estimate conducted by the Greenhouse Gas R&D Program of the International Energy Agency, there are 188 trillion m^3^ of technically recoverable shale gas globally. This could facilitate the potential storage of 740 Gt of CO_2_ in shales [5]. In addition, 50 trillion m^3^ of methane could be recovered using CO_2_-enhanced coal bed methane recovery (CO_2_-ECBM) technology, and 488 Gt of CO_2_ could be stored in the coal beds during this process [6].

The bituminous coal, anthracite, and shale samples used in this study were collected from the Ordos Basin. The Ordos Basin is the second-largest sedimentary basin in China, and it contains resources such as coal bed methane, coal, shale gas, and oil shale [7]. A sample of marine-continental transitional facies shale was collected from a location with a complex depositional environment. In the location where the shale samples were collected, the vertical profile consists of a variety of lithologies, including sandstone, siltstone, shale, and coal [8]. A joint study of coal beds and shales is an important inspiration for promoting unconventional natural gas extraction, as well as the development of CO_2_ geologic storage in this coal-bearing rock series.

At target depths of more than 800 m for EGR, reservoir conditions can reach supercritical conditions for CO_2_, i.e., 31.1 °C and 7.38 MPa. According to previous studies, supercritical CO_2_ (Sc-CO_2_) injection can cause the dissolution of silicate minerals, e.g., illite, kaolinite, and biotite, leading to alteration of pore structure [9,10,11,12]. Changes in pore heterogeneity can be studied using fractal theory. It has been found that the interactions between Sc-CO_2_ and coal can enhance structural complexity and pore roughness [13,14]. Many scholars have different ideas regarding how the characteristics of different types of pores (e.g., volume and surface area) vary. It has been argued by many scholars that the formation of new pores is due to the transformation of small pores into large pores. This results in a significant increase in the proportion of macropores in coal [11,15,16,17,18]. Zhang et al. [19] found that for coals, ScCO_2_–H_2_O exposure has a slight effect on micropores (<2 nm) and a remarkable effect on mesopores (2–50 nm). In particular, the surface area, volume, and average pore size of mesopores in coals decrease after exposure. Kang et al. [20] investigated the decrease in pore volume after supercritical CO_2_ extraction. They found that this effect was more significant for pore widths of 0.35–5 nm and for low-rank to medium-rank coals. Cheng et al. [18] believed that the increases in the porosity and pore volume of bituminous coal caused by CO_2_ were mainly concentrated on mesopores (<10 nm) and macropores (10–100 nm). Because these studies treated samples with Sc-CO_2_ at different pressures, temperatures, and durations, the pore size ranges they focused on also varied. It is necessary to conduct consistent Sc-CO_2_ treatments on shale and coal to study the resulting changes in pore structure.

It is costly to capture and separate CO_2_. Taking coal-fired power plants as an example, the cost of separating CO_2_ from flue gas and subsequent purification can increase power costs by approximately 75% [21], which makes the practical application of CO_2_-EGR challenging. Direct injection of industrial waste gases, including CO_2_ and NO, into coal beds has recently attracted attention because it reduces the denitrification cost [22,23]. Prolonged exposure to NO decreases the number of oxygen-containing functional groups, while markedly increasing amine content. These changes in surface chemical properties suggest that the coal matrix can chemically adsorb NO, enabling stable storage within targeted coal reservoirs [24]. Moreover, the amine groups on pore surfaces have been utilized to improve the selective adsorption of CO_2_ based on the electron donor-acceptor interactions between the amines and CO_2_ [25]. However, CO_2_ and NO have greater adsorption affinity for coal and shale than N_2_ and CH_4_, and their injection may trigger a dramatic decrease in reservoir permeability [26,27]. Partial mixing of N_2_ can be used to reduce matrix swelling and thereby increase the injection rate during the EGR process [28,29]. Thus, in this study, the CO_2_-rich industrial waste gas (CO_2_-rich IWG) utilized was a mixture of CO_2_, NO, and N_2_. The current research on the comprehensive impact of CO_2_-rich IWG on the pore structures of coal and shale remains limited. This study aims to advance our understanding of this process.

In summary, in this study we systematically investigated the effect of CO_2_-rich IWG on the pore structures of coal and shale. In this study, we collected three coal samples with different maturities, namely, lignite, bituminous coal, and anthracite samples, and one shale sample. The pore volume (PV), specific surface area (SSA), and pore size distribution (PSD) of micropores, mesopores, and macropores were analyzed before and after treatment with CO_2_-rich IWG. The effects of pore structure changes on CH_4_ adsorption capacity and pore connectivity were also explored. This work provides a reference for the sequestration of CO_2_-rich IWG in the studied coal-bearing rock series.

## 2. Samples and Methodology

### 2.1. Typical Rock Samples

**Figure 1:**
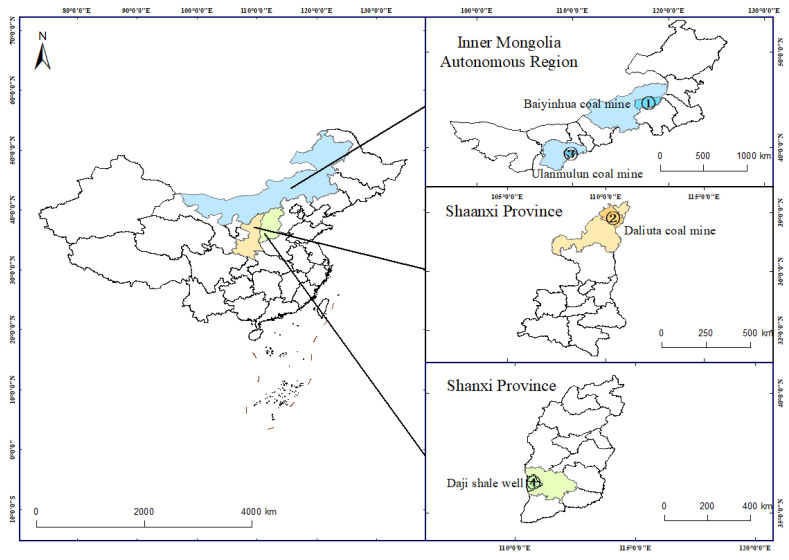
Map showing the sampling locations.

Three coal samples with different ranks (i.e., lignite, bituminous coal, and anthracite) were chosen according to ISO 11760:2018 [30]. These samples represent a wide range of coals from low to medium to high ranks. As shown in Figure 1, the lignite and anthracite samples were collected from the Baiyinhua coal mine in the Xilingol League and the Ulanmulun coal mine in Ordos, Inner Mongolia Autonomous Region, China, respectively. The bituminous coal sample was collected from the Daliuta coal mine in Yulin, Shaanxi Province, China, and the shale sample was collected from the Daji shale well in Linfen, Shanxi Province, China. The lignite, bituminous coal, and anthracite coal samples and the shale sample were numbered 1 to 4, respectively, and their properties are shown in Table 1.

### 2.2. CO_2_-Rich IWG Treatment Process

The CO_2_-rich IWG treatment was conducted in geochemical reactors. Four 200 mL reactors were prepared, and their temperatures and pressures were monitored in real-time using high-precision transducers. The samples were placed in their respective reactors. A booster pump was utilized to achieve high pressure, and a high-temperature system was achieved using an oil bath. The experimental temperature and pressure were selected to be 110 °C and 13.0 MPa based on the temperature and burial depth of the subsurface constant temperature zone, the average geothermal gradient, and the average pressure gradient at the sampling location. The CO_2_-rich IWG had a mass fraction of CO_2_:N_2_:NO= 70:25:5 and a pressure of 4.5 MPa.

**Figure 2:**
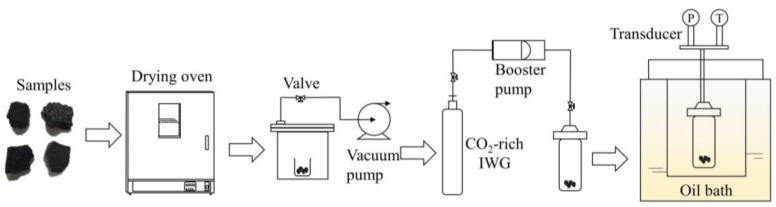
Schematic diagram of the CO_2_-rich IWG treatment process.

We cut the samples into approximately cubic shapes of 3–4 cm^3^ before conducting the CO_2_-rich IWG treatment, as large coal blocks may lead to a long experimental reaction time. The CO_2_-rich IWG treatment process involved six steps (Figure 2).

(1) The samples were heated in a drying oven at 100 °C for at least 2 h to remove the water in the samples.
(2) The dried samples were placed in a sealed container and degassed using a vacuum pump for at least 8 h to remove any residual gases in the samples.
(3) CO_2_-rich IWG was injected into the reactors using a booster pump until the pressure transducer indicated that the set value of 8.5 ± 0.5 MPa had been reached.
(4) An airtightness check of the reactors was conducted. The oil bath heating system was turned on and the reactors were placed in silicone oil until the temperature stabilized at 110 ± 0.5 °C and the pressure stabilized at 12.5 ± 0.5 MPa.
(5) The samples were subjected to continuous treatment in the reactors for 14 days at a temperature and gas pressure of 110 ± 0.5 °C and 12.5 ± 0.5 MPa. Then, valves were opened to discharge residual gas. The treated coal samples were packed in sealed bags for further tests.
(6) Via the above experimental procedure, eight groups of samples were prepared for subsequent experiments, including four untreated samples and four CO_2_-rich IWG-treated samples.

### 2.3. Gravimetric Isothermal Adsorption

The gravimetric adsorption instrument utilized was a magnetic suspension mass method isothermal adsorption instrument (ISO-SORP^®^STATIC, Rubolab GmbH, Wertheim, Germany). The magnetic suspension balance utilized has an accuracy of 0.01 mg and a maximum pressure and temperature of 35 MPa and 200 °C, respectively. The temperature of the sample container was controlled using a heating oil-bath cycle with an accuracy of 0.01 °C. The gravimetric adsorption measurement involved three steps: sample preprocessing, buoyancy measurement, and adsorption measurement [31].

#### 2.3.1. Sample Preprocessing

The samples were crushed and sieved, and 60- to 80-mesh particles (0.180–0.250 mm) were selected for use. Then, the samples were dried for 3 h at 70 °C in He with a purity of 99.999%, followed by drying at 75 °C under vacuum for 2 days.

#### 2.3.2. Buoyancy Measurement

According to the experimental principle of the magnetic suspension mass method isothermal adsorption instrument, the mass balance equation is as follows [32]:

(1) $$m_{c+s}=m_{1}+\rho_{He}V_{c+s},$$

where $m_{c+s}$ is the mass of the sample container and sample (g); $V_{c+s}$ is the volume of the sample container and sample (g/cm^3^); $m_{1}$ is the mass measured by the magnetic suspension balance (g); and $\rho_{He}$ is the density of the He (g/cm^3^). The values of $m_{1}$ and $\rho_{He}$ were recorded by the data acquisition computer.

Five groups of data for $m_{1}$ and $\rho_{He}$ were acquired under 1, 2, 3, 4, and 5 MPa. The $m_{c+s}$ and $V_{c+s}$ at the test temperature were determined using the linear fitting method.

#### 2.3.3. Adsorption Measurement

The mass balance equation is as follows:

(2) $$m_{c+s}+m_{abs}=m_{2}+\rho_{CH_{4}}V_{c+s},$$

where $m_{abs}$ is the mass of the absorbed CH_4_ (g); $m_{2}$ is the mass measured by the magnetic suspension balance (g); $\rho_{CH_{4}}$ is the density of the CH_4_ (g/cm^3^); and $m_{c+s}$ and $V_{c+s}$ are determined using Equation (1).

The methane adsorption capacity equation is as follows:

(3) $$V=(V_{CH_{4}}m_{abs}/M_{CH_{4}})/m_{s},$$

(4) $$m_{s}=m_{c+s}-m_{c},$$

where V is the methane adsorption capacity (cm^3^/g); $V_{CH_{4}}$ is the volume of 1 mol of ideal CH_4_ (cm^3^/mol); $M_{CH_{4}}$ is the molar mass of CH_4_ (g/mol); and $m_{s}$ and $m_{c}$ are the masses of the sample and sample container, respectively.

In the adsorption measurement, the experimental temperature was set to 20 °C (293.75 K), and 19 pressure points were measured, namely, 0.50 MPa, 1.00 MPa, 2.00 MPa, 3.00 MPa, 4.00 MPa, 5.00 MPa, 6.00 MPa, 7.00 MPa, 8.00 MPa, 9.00 MPa, 10.00 MPa, 12.00 MPa, 14.00 MPa, 16.00 MPa, 18.00 MPa, 20.00 MPa, 24.00 MPa, 28.00 MPa, and 32.00 MPa. The adsorption equilibrium conditions were determined from the sample mass variation, which should be less than 0.001 g within 10 min.

### 2.4. Pore Size Characterization

The pore classification criteria recommended by the International Union of Pure and Applied Chemistry (IUPAC) were used in this study. The pores were categorized as micropores (diameter of <2 nm), mesopores (diameter of 2–50 nm), and macropores (diameter of >50 nm) [33].

#### 2.4.1. Low-Temperature CO_2_ Adsorption

CO_2_ has a molecular dynamics diameter of 0.33 nm, which is more accessible to the ultra-micropores in the samples [34]. The CO_2_ adsorption test was conducted using an Autosorb-iQ3 instrument (Quantachrome Instruments, Boynton Beach, FL, USA). In the test, the particle size of the samples was 60–80 mesh (0.180–0.250 mm), CO_2_ was used as the adsorbent, and the analysis bath temperature was 273.15 K.

The SSA of the micropores and the pore volume were calculated using the Dubinin–Radushkevich (DR) method, and the pore size distribution within the range of 0.35–1.50 nm was analyzed using nonlocal density functional theory (NLDFT).

Molecular adsorption with nonlocal density functional theory (NLDFT) is one of the most widely used methods today for nanoscale-resolution PSD. The NLDFT method uses classical fluid density functional theory to construct the adsorption isotherms in ideal pore geometries. The PSD result is found by solving an adsorption integral equation (Equation (5), the first term). Since this is an ill-posed problem, regularization techniques are used to stabilize the solution (Equation (5), the second term) [35]. Thus, the PSD solution will be dependent upon the chosen regularization parameter, λ (also known as the smoothing parameter). In this equation, $N_{\exp}$ is the experimental gas adsorption, $N_{NLDFT}$ represents the theoretical gas isotherms assuming an ideal pore geometry such as slit pore and cylindrical, PSD is the pore size distribution, $\frac{P}{P_{0}}$ is the pressure ratio with respect to the gas saturation pressure, and D is the pore diameter.

(5) $$N_{\exp}(\frac{P}{P_{0}})=\int N_{NLDFT}(\frac{P}{P_{0}},D)PSD(D)dD+\lambda \int [PSD^{N}(D){]}^{2}dD$$

#### 2.4.2. Low-Temperature N_2_ Adsorption

The N_2_ adsorption test was conducted using an Autosorb-iQ3 instrument (Quantachrome Instruments, USA). In the test, the particle size of the samples was 45–60 mesh (0.250–0.355 mm), and the N_2_ adsorption/desorption data were obtained at 77.0 K.

The pore SSA was calculated using the Brunauer–Emmett–Teller (BET) equation, and the PV was calculated using the Barrett–Joyner–Halenda (BJH) model. The mesopore size within the range of 2–50 nm was analyzed using nonlocal density functional theory (NLDFT).

#### 2.4.3. Mercury Intrusion Porosimetry

Mercury intrusion porosimetry (MIP) was performed using a MicroActive AutoPore V 9600 (Micromeritics Instrument Corporation, Norcross, GA, USA). The samples used for the mercury intrusion porosimetry tests were small blocks with volumes of 3–4 cm^3^. The pressure in the mercury intrusion porosimetry tests ranged from 0.0071 to 275.05 MPa to avoid high-pressure effects that may distort data for smaller pores [36]. The pore size was determined using the Washburn equation (Equation (6)) [37], in which the pores are assumed to be cylindrical, and the specific surface areas of the pores can be calculated using Equation (7) [38].

(6) $$P_{Hg}=\frac{2\gamma \cos\theta }{r},$$

where $P_{Hg}$ is the mercury intrusion pressure (MPa); γ is the mercury surface tension, which is 0.48 N/m; θ is the contact angle between the mercury and sample, which is 130°; and *r* is the pore radius (µm).

(7) $$S=-\frac{1}{\gamma \cos\theta }\int_{0}^{V}P_{Hg}dV,$$

where *S* is the specific surface area of the pore (m^2^/g) and *V* is the cumulative intrusion volume (cm^3^/g).

## 3. Results and Discussion

This section discusses the pore structure characteristics of the micropores, mesopores, and macropores before and after the CO_2_-rich IWG treatment. The effect of the micropore structure changes on adsorption capacity was investigated via CH_4_ isothermal adsorption. The impact of the macropore structure changes on gas transport capacity was analyzed based on pore connectivity.

The test results revealed that the PV and SSA of the shale were sometimes several orders of magnitude smaller than those of the coals, which was mainly determined by their geological origins. Coal is formed from plant materials through dehydration, deoxidation, and carbonization under low oxygen, high temperature, and high-pressure conditions. Therefore, coal has a high original organic matter content, which provides sufficient material for the development of extensive pore structures in coal [39]. Shale is a sedimentary rock composed of clay, mud particles, and small amounts of organic matter that have been compressed and hardened over a long time period. Shale usually has a low organic matter content, such as kerogen, which is usually dispersed within the mineral matrix. The limited organic content restricts the number and size of the pores in shale [40,41].

**Figure 3:**
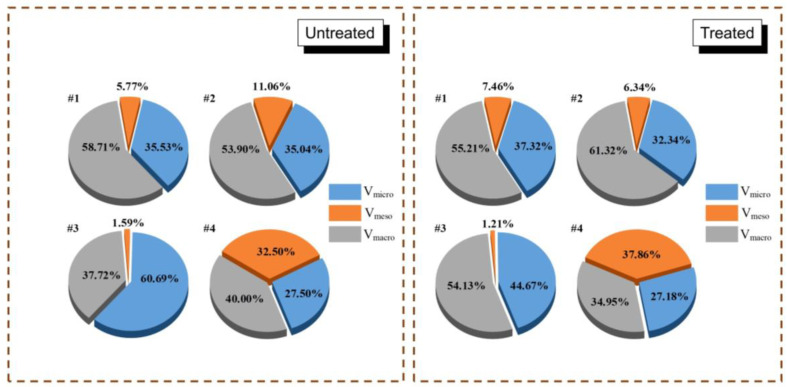
Pore volume distribution of four samples before and after CO_2_-rich IWG treatment. *Note:* #1—lignite; #2—bituminous coal; #3—anthracite; and #4—shale.

Figure 3 shows the pore volume distribution of four samples before and after CO_2_-rich IWG treatment. Both lignite and bituminous coal exhibit similar distributions before and after treatment, dominated by macropores, followed by micropores, with mesopores having the smallest proportion. Anthracite had the highest proportion of micropores before treatment, while after treatment macropores became the dominant fraction. Shale shows a special pore structure distribution, with a similar proportion among the three pore types. Before treatment, macropores were dominant in shale, but after treatment mesopores became the main pore type.

### 3.1. Evolution of Micropore Structure Characteristics

**Figure 4:**
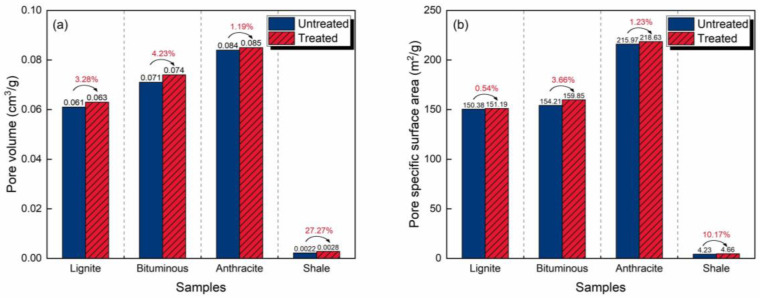
Pore structure characteristics and changes analyzed by DR method resulting from the CO_2_-rich IWG treatment of the four samples: (**a**) pore volume and (**b**) pore specific surface area.

Figure 4 illustrates the characteristics of the pore structures before and after CO_2_-rich IWG treatment and the changes. Before the treatment, the PV and SSA of the coals increased with increasing coal rank. The anthracite had the largest PV (0.084 cm^3^/g) and SSA (215.97 m^2^/g), while the lignite had the smallest PV (0.061 cm^3^/g) and SSA (150.38 m^2^/g). After the treatment, this trend remained the same. The PV and SSA of the anthracite increased to 0.085 cm^3^/g and 218.63 m^2^/g, and those of the lignite increased to 0.063 cm^3^/g and 151.19 m^2^/g, respectively. Additionally, the PV of the shale was one order of magnitude smaller than those of the coals, and its SSA was two orders of magnitude smaller.

The enhancements in the coals were slight, with PV change rates ranging from 1.19% (anthracite) to 4.23% (bituminous coal) and SSA change rates ranging from 0.54% (lignite) to 3.66% (bituminous coal). Comparatively, the effect of the treatment on the shale was more obvious, with improvements in PV and SSA reaching 27.27% and 10.17%, respectively. Coal and shale, as materials composed of macromolecules, are subjected to structural reorganization under CO_2_-rich IWG treatment [13,42]. For example, intermolecular chemical bonds are broken and minerals are decomposed, thereby enhancing pore development.

**Figure 5:**
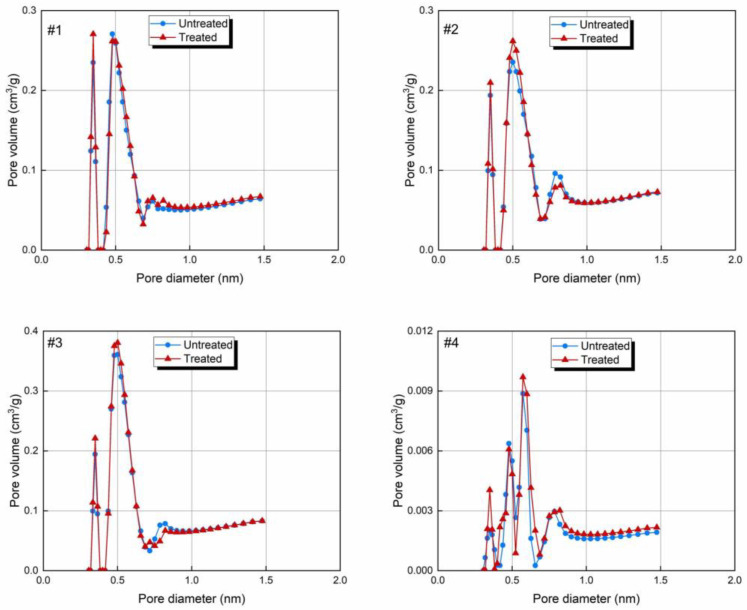
The pore size distributions of the micropores analyzed by NLDFT method before and after CO_2_-rich IWG treatment. *Note:* #1—lignite; #2—bituminous coal; #3—anthracite; and #4—shale.

Figure 5 shows the size distributions of the micropores under the conditions before and after the CO_2_-rich IWG treatment. The PSD curves of the coals and shale differ significantly, with a double peak for the coal samples and a triple peak for the shale. The coals and shale had similar pore volume distributions, which were primarily concentrated within the 0.4–0.7 nm pore size range.

There was an increase in the first main peak on all of the treated curves, which was the primary reason for the improvement in PV. The pore diameter corresponding to the main peak remained almost unchanged, while both the PV and SSA increased (Figure 4). This indicates that the diameter of the main pores did not become larger or smaller, and the length or number increased. The expansion was due to the fact that the CO_2_-rich IWG treatment dissolved the mineral matrix within the pores, thereby opening previously blocked pores. The increase in the SSA can be attributed to the removal of surface minerals, exposing more adsorption sites and enhancing the effective specific surface area.

### 3.2. Evolution of Mesopore Structure Characteristics

**Figure 6:**
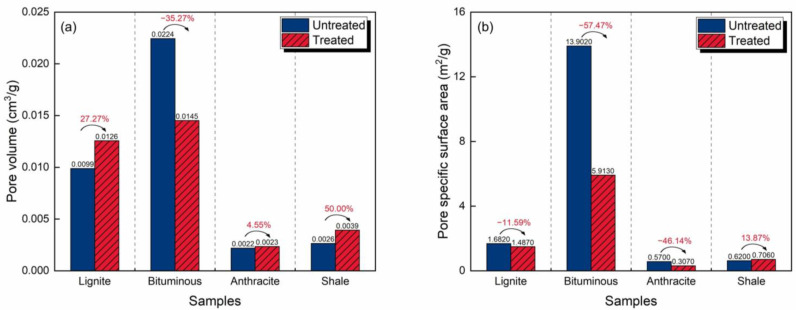
Pore structure characteristics and changes analyzed by BET and BJH methods with CO_2_-rich IWG treatment for the four samples: (**a**) pore volume and (**b**) pore specific surface area.

The pore structure characteristics of the samples with and without CO_2_-rich IWG treatment, and their changes, are illustrated in Figure 6. The relationships between coal rank and PV and SSA exhibited inverted V-shaped trends before and after treatment. The bituminous coal had the largest PV and SSA, while the anthracite coal had the smallest PV and SSA. The PV and SSA of the shale were intermediate between the three types of coal, with values slightly larger than those of the anthracite and smaller than those of the lignite.

The PVs of all of the samples, except the bituminous coal, were larger after the treatment. The PVs of the shale and lignite increased remarkably by 50.00% and 27.27%, respectively, while that of the bituminous coal decreased by 35.27%. That of the anthracite decreased slightly by 4.55%. Regarding SSA, the SSAs of all of the samples, except for the shale, were lower after the treatment. The bituminous and anthracite coals were significantly affected, with reductions of 57.47% and 46.14%, respectively. The PVs of the lignite and anthracite increased, but their SSAs decreased. This will be explained in detail based on the PSD curves.

**Figure 7:**
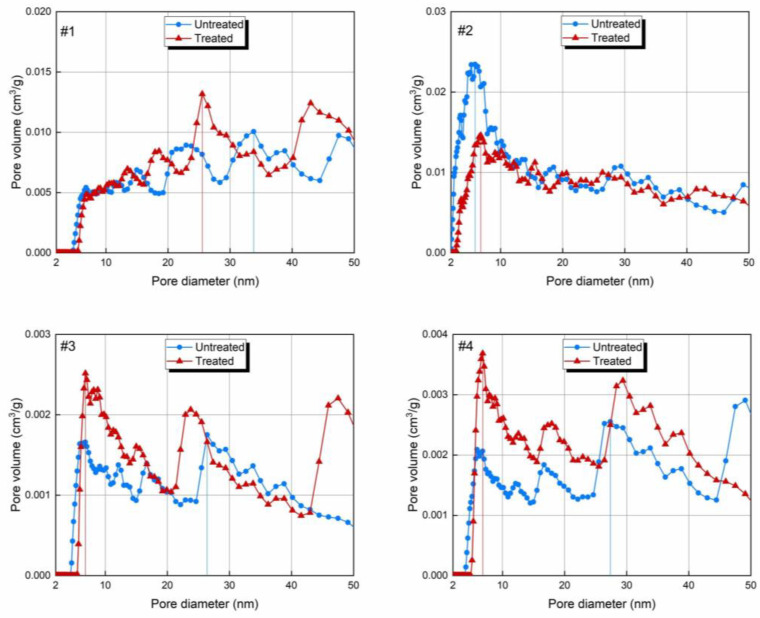
Pore size distribution of mesopores analyzed by NLDFT before and after CO_2_-rich IWG treatment. *Note:* #1—lignite; #2—bituminous coal; #3—anthracite; and #4—shale. The vertical line indicates the maximum value.

The PSD is presented in Figure 7. The PSD of the bituminous coal exhibits a single prominent peak in the pore diameter range of 2–10 nm, while the other three samples exhibit two–three distinct peaks. This trend is consistent with the pore characteristics observed after the treatment. By comparing the PSDs before and after the treatment, it was found that the maximum pore volume of the bituminous coal decreased, while the peak values of the other samples increased. As marked by the vertical lines in Figure 7, except for the bituminous coal, the pore diameter corresponding to the maximum pore volume after treatment was smaller than before the treatment, indicating that the major pore size decreased, which was favorable for gas adsorption. The decrease in the SSA of the bituminous coal was more pronounced than the decrease in PV and this was due to the enlargement of the main pores, as smaller pores make a greater contribution to SSA. The increases in the PVs and decreases in the SSAs of the lignite and anthracite can be attributed to the formation of larger pores within the samples, as evidenced by the growth of the PSD curve in the 40–50 nm pore diameter range. These larger pores contributed to the increase in the total PV; however, due to their smaller SSA per unit volume, they led to a decrease in the overall SSA.

### 3.3. Evolution of Macropore Structure Characteristics

**Figure 8:**
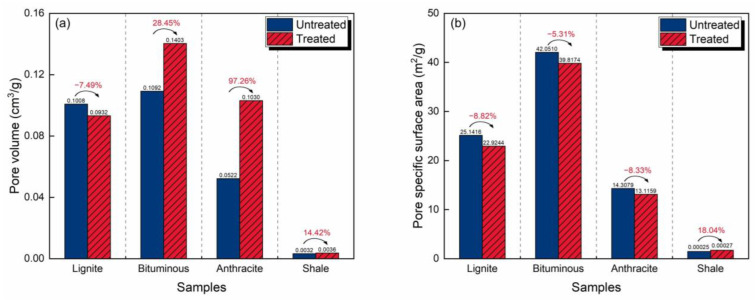
Pore structure characteristics and changes caused by the CO_2_-rich IWG treatment of the four samples, analyzed by Washburn equation: (**a**) pore volume and (**b**) pore specific surface area.

The pore structure characteristics of the four samples and their changes under CO_2_-rich IWG treatment are shown in Figure 8. Before the treatment, PV and SSA exhibited inverted V-shaped trends as coal rank increased. The bituminous coal had the largest PV and SSA, reaching 0.1092 cm^3^/g and 42.0510 m^2^/g, respectively. The anthracite had the smallest PV and SSA, with values of 0.0522 cm^3^/g and 14.3079 m^2^/g, respectively. The PV of the shale was two orders of magnitude smaller than that of the coals, and the SSA was five orders of magnitude smaller.

After the treatment, the relationships between the pore structure characteristics and coal rank still exhibited inverted V-shaped trends. The coal with the smallest PV changed from anthracite to lignite, with a value of 0.0932 cm^3^/g. The anthracite had the smallest SSA of 13.1159 m^2^/g. The bituminous coal still had the largest PV and SSA, with values of 0.1403 cm^3^/g and 39.8714 m^2^/g, respectively.

The PVs of all of the samples, except the lignite, were larger after CO_2_-rich IWG treatment. The anthracite experienced the largest increase (97.26%), while that of the lignite decreased by 7.49%. The reduction in the PV of the lignite is the reason why it became the coal sample with the smallest PV after the treatment. The SSAs of the coal samples decreased by 5.31–8.82%. In contrast, that of the shale sample increased by the greatest percentage (18.04%).

**Figure 9:**
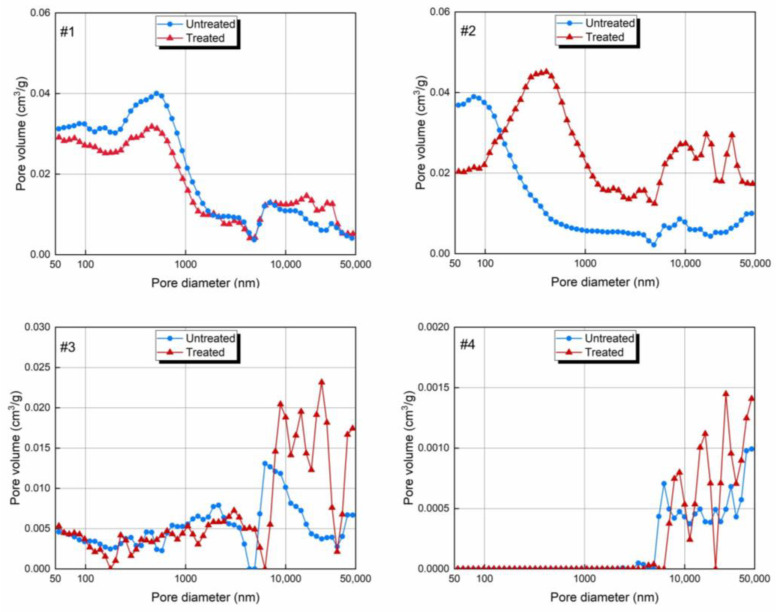
Pore size distributions of the macropores before and after the CO_2_-rich IWG treatment, analyzed by Washburn equation. *Note:* #1—lignite; #2—bituminous coal; #3—anthracite; #4—shale.

The PSDs of the macropores are shown in Figure 9. The PSDs exhibited the same characteristics before and after the CO_2_-rich IWG treatment. Lignite and bituminous coal had a distinct peak and a fluctuating segment, while anthracite and shale had a segment of peaks. The peak of the lignite occurred in the pore size range of 100–1000 nm, and the peak of the bituminous coal occurred at 50–1000 nm. The peaks of the anthracite and shale occurred in the range of 10–50 μm. The peaks and fluctuation segments indicate the presence of pores with corresponding sizes in the sample.

By comparing the PSD curves before and after the treatment, it was found that only the peak of the lignite exhibited a certain degree of reduction. This is the reason for the reduction in the PV of the lignite. The peaks of the remaining three samples all increased, and the order of the magnitudes of the changes was anthracite > bituminous coal > shale. This is consistent with the changes in PV (Figure 8). It should be noted that a leftward shift in the peak of the bituminous coal indicates that the PV of the bituminous coal increased at the same time as its pore diameter increased. The fluctuation segments for all of the samples did not vary. The peaks of the fluctuation segments all increased. It was found that the fluctuations in the fluctuation segments become greater, indicating that the pore structures of the samples became more complex.

### 3.4. Effects of Changes on Adsorption Capacity Within Micropores and Mesopores

Micropores are small in size but large in number and have a huge specific surface area. Thus, they have strong storage capacity for gases and are the most important gas storage sites in reservoirs. Mesopores act as bridges in pore structure, forming a transition region between micropores and macropores. Mesopores serve as buffer pores for monolayer gas adsorption during initial storage or desorption [43,44]. An intensive, detailed understanding of the structure of micropores and mesopores is important for understanding the gas storage capacities of coal and shale.

#### 3.4.1. CH_4_ Adsorption Isotherms

**Figure 10:**
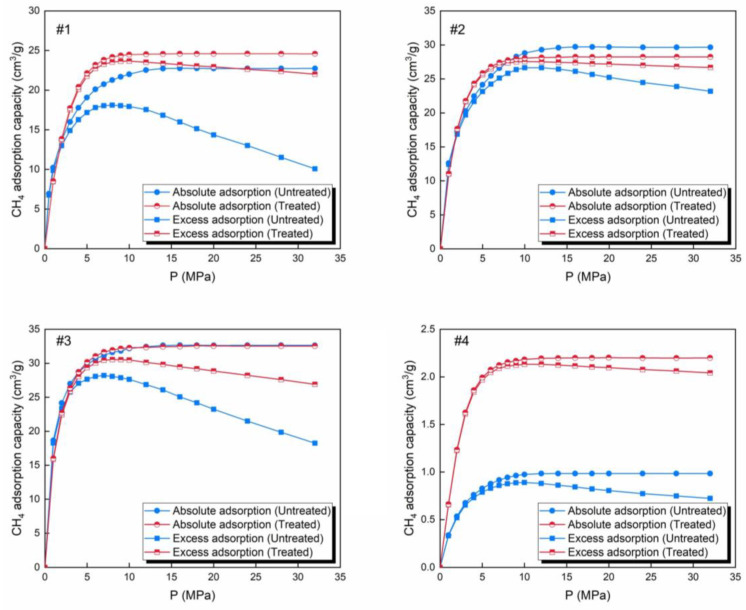
CH_4_ adsorption isotherms at 293.15 K for the samples before and after CO_2_-rich IWG treatment. *Note:* #1—lignite; #2—bituminous coal; #3—anthracite; #4—shale.

The CH_4_ adsorption isotherms at 293.15 K are shown in Figure 10, and all of the samples have two types of curve; namely, absolute adsorption and excess adsorption. Absolute adsorption represents the actual amount of gas adsorbed by the adsorbate, while excess adsorption is the absolute adsorption minus the amount of gas that exists in the gas phase in the adsorbate pores.

The order of CH_4_ excess and absolute adsorption capacity for the samples is as follows: anthracite > bituminous coal > lignite > shale, both before and after treatment. The excess adsorption capacities of the samples were significantly enhanced because the treatment process cleaned the impurities from the pores in the samples, altered the surface properties of the adsorbate, and improved its gas adsorption capacity. At pressures of <5 MPa, the gas filled the pore surfaces, and the amount of adsorption increased rapidly. As the pressure increased, the number of molecules increased and the gas molecules began to stack up. This portion of the gas was not adsorbed, but it still occupied space, so a difference appeared between the absolute adsorption and the excess adsorption. This difference increased as the gas continued to accumulate. It was observed that for the coal samples, the difference decreased significantly, while for the shale, it was slightly lower after the CO_2_-rich IWG treatment. The improvement in excess adsorption capacity reduced the amount of gas not adsorbed in the pores, thereby narrowing the difference. The PV of the shale was small, so the variation in the gas phase molecules within the pores was limited.

#### 3.4.2. Relationships Between Adsorption Capacity and Micropores and Mesopores

**Figure 11:**
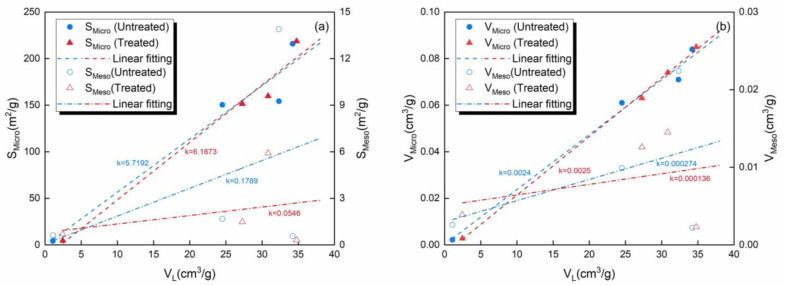
Relationships between the (**a**) SSA and *V_L_*, (**b**) PV and *V_L_* of the micropores and mesopores before and after CO_2_-rich IWG treatment.

The changes in the absolute CH_4_ adsorption capacities of the samples with pressure were fitted using the Langmuir adsorption model. The fitting parameters are shown in Table 2. The Langmuir volume (*V_L_*) is presented in the form of scatter points in Figure 11. Linear fitting was conducted to explore the influences of the SSA and PV of the micropores and mesopores on *V_L_*.

The SSA and PV of the micropores and mesopores exhibited positive correlations with *V_L_*. The string correlation between the micropores and adsorption capacity indicates that the micropores were the main space for gas adsorption. SSA influences adsorption capacity by affecting the sites of surface adsorption, and it influences PV by determining the space-filling capacity of molecules within the pores. The CO_2_-rich IWG treatment resulted in an increase in the influence of micropores on gas adsorption capacity and a decrease in that of mesopores.

The ranking of absolute adsorption capacity (Figure 10) aligns with the SSA ranking of the micropores, i.e., anthracite > bituminous > lignite > shale, highlighting the dominant role of the SSA of the micropores. However, the SSA of the micropores cannot fully explain the variation in the CH_4_ adsorption capacity. The excess adsorption capacity increased, primarily due to the increase in the SSA of the micropores in the four samples, which provided more adsorption sites. However, the extent of the excess adsorption capacity did not directly align with the increase in the SSA of the micropores. The order of the excess adsorption capacity was shale > lignite > anthracite > bituminous coal, while the order of SSA was shale > bituminous coal > anthracite > lignite. The reasons for this phenomenon need to be analyzed in combination with other parameters. The bituminous coal was the only sample in which the main pore diameter of the mesopores increased, which was unfavorable for adsorption. Additionally, the decreases in the SSA and PV of the mesopores in the bituminous coal were the largest. These two unfavorable factors resulted in the bituminous coal experiencing the smallest increase in excess adsorption capacity. The changes in the three parameters, other than SSA, of the micropores of the lignite promoted adsorption more than those in the anthracite, which was the reason the increase in the excess adsorption capacity of the lignite was greater than that of the anthracite.

### 3.5. Pore Connectivity Within Macropores

Macropores are important pathways for gas transportation in geologic reservoirs [45]. The pores in coal and shale are highly complex, with varying pore size and throat widths. In some pores, the throat width is relatively small, and once mercury is injected under pressure, it cannot be completely expelled. This results in a hysteresis between mercury intrusion and extrusion. Therefore, hysteresis to some extent represents the connectivity of the pore network [46]. Pores can be categorized into effectively interconnected pores and non-effectively interconnected pores with extremely narrow throats based on their connectivity [47]. The former mainly affects fluid transportation. The better the connectivity is, the smoother the fluid flow is. The volumes of the non-effectively and effectively interconnected pores can be calculated from cumulative mercury intrusion and extrusion volumes using the following equations [47]:

(8) $$V_{Clo}=V_{Eje,des},$$

(9) $$V_{Total}=V_{Inj,des}=V_{Eje,sta}=V_{Inter}+V_{Clo},$$

where $V_{Clo}$ is the volume of non-effectively interconnected pores (cm^3^/g); $V_{Inter}$ is the volume of effectively interconnected pores (cm^3^/g); $V_{Total}$ is the total pore volume of mercury intrusion porosimetry (cm^3^/g); $V_{Inj,des}$ is the pore volume at the end of mercury intrusion (cm^3^/g); $V_{Eje,sta}$ is the pore volume at the start of mercury ejection (cm^3^/g); and $V_{Eje,des}$ is the pore volume at the end of mercury ejection (cm^3^/g).

The volumes of effectively interconnected pores and non-effectively interconnected pores before and after CO_2_-rich IWG treatment are presented in Table 3. The volumes of the effectively interconnected pores in the lignite and anthracite decreased, while those in the bituminous coal and shale increased. The lignite experienced the largest change in effectively interconnected pore volume (13.64%), while the other three samples experienced changes ranging from 5.00% to 5.97%. This suggests that the CO_2_-rich IWG treatment had a greater impact on the lignite and little effect on the other samples. The CO_2_-rich IWG treatment significantly changed the volumes of the non-effectively interconnected pores in the samples. The rate of increase of the non-effectively interconnected pores increased with increasing coal rank. In particular, for the anthracite, the rate of increase of non-effectively interconnected pores reached 177.21%, which was the main contributor to the increase in its PV.

In summary, the CO_2_-rich IWG treatment affected macropores in the different samples in different ways. It had a negative impact on both the PV and connectivity of the lignite. For the bituminous coal, the CO_2_-rich IWG treatment increased its PV by enlarging pore diameter and improving connectivity. For the anthracite, the CO_2_-rich IWG treatment greatly increased pore volume, primarily for non-effectively interconnected pores. For the shale, the CO_2_-rich IWG treatment comprehensively improved the parameter characteristics of macropores in all aspects.

### 3.6. Mechanism of CO_2_-Rich IWG Treatment

It is essential to explore the interactions of CO_2_-rich IWG components with coals and shale, as well as how these interactions affect the physical properties and adsorption characteristics of coal. The various components of CO_2_-rich IWG have different functions. CO_2_ modifies pore structure by promoting the dissolution of minerals. NO acts as a chemically reactive gas and reacts with minerals or organic matter in coal. N_2_ serves as an inert gas in pore spaces and maintains the pressure and diffusion equilibrium of the environment. Although the experiments in this study were conducted under dry conditions, it is difficult to completely remove all moisture from samples. Therefore, the reactions involving water discussed in this section remain relevant and provide useful insights into the related mechanisms.

As a reactive gas, CO_2_ dissolves in water to form carbonic acid (H_2_CO_3_) and undergoes the following further dissociation reactions:

(10) $$H_{2}CO_{3}\to HCO_{3}{\,}^{-}+H^{+}.$$

(11) $$HCO_{3}{\,}^{-}\to CO_{3}{\,}^{2-}+H^{+}.$$

The hydrogen ions (H^+^) that are produced decrease the pH of the water and create an acidic environment. Carbonate minerals can be dissolved in an acidic environment and the following reaction occurs:

(12) $$CaCO_{3}+H^{+}\to Ca^{2+}+HCO_{3}{\,}^{-}$$

**Figure 12:**
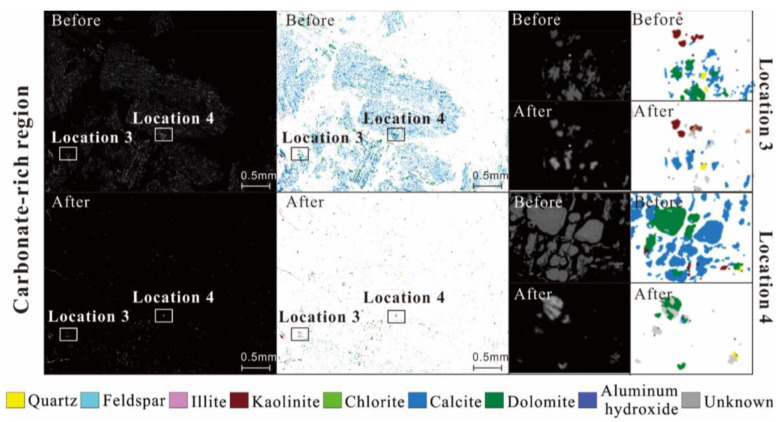
SEM back-scattered electron images and mineral identification images showing the mineral distribution of carbonate-rich regions before and after Sc-CO_2_ treatment. *Note:* Taken with permission from [48].

**Figure 13:**
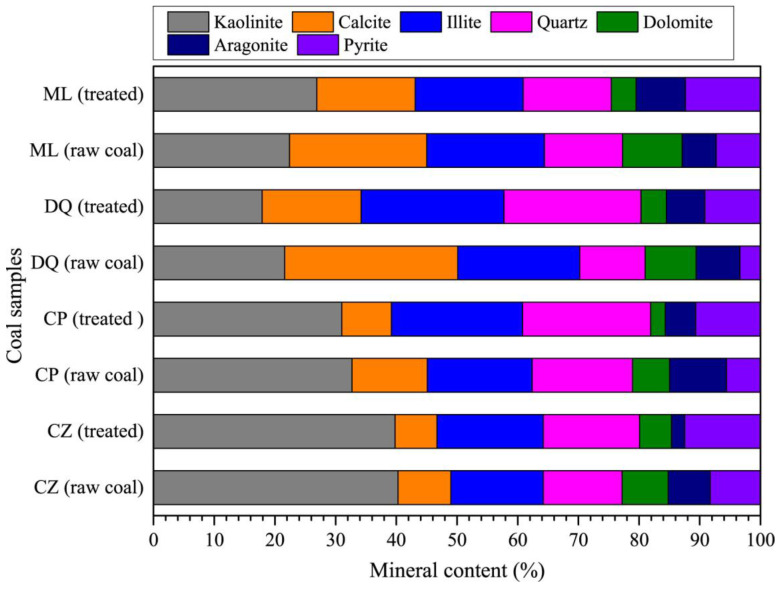
Alterations in mineral content within coal after Sc-CO_2_ treatment analyzed by X-ray diffraction (XRD). *Note:* Samples ML and DQ are bituminous coal. Samples CP and CZ are anthracite. Taken with permission from [13].

By examining scanning electron microscope (SEM) images of the coal treated with CO_2_, we found that significant changes had occurred on the surfaces of the carbonate-rich samples. A large amount of carbonates (calcite and dolomite) had dissolved and the surface became a honeycomb-like structure, as shown in Figure 12 [48]. The results of X-ray diffraction (XRD) analysis also support the conclusion that both dolomite and calcite had undergone dissolution, as shown in Figure 13 [13]. In shale, the situation was slightly different, as CO_2_ can only dissolve calcite but not dolomite [49]. Sc-CO_2_ also affects the functional groups of the sample. Sc-CO_2_ has a strong extraction capacity for hydrocarbons, and approximately 80% of aliphatic structures are extracted from coal macromolecules [50]. The ratio of aromatic to aliphatic groups was used to measure aromaticity. A decrease in intensities of aliphatic stretching bands was observed, with no significant changes in the aromatic stretching band in the FIRT spectra of coal after Sc-CO_2_ treatment [51]. This led to an increase in the degree of aromaticity and a stronger affinity for CO_2_ adsorption. In addition, mineral dissolution due to Sc-CO_2_ injection reduced the grain-to-grain contacts in the reservoir rock and the bond energy of the pore structure [52,53]. Not only the non-covalent bonding energy was reduced, but also the bond angle energy and torsion energy were changed, leading to the alteration of the macromolecular structure, thereby inducing changes in the pore structure [54,55].

Chemical adsorption is the primary interaction mechanism between NO and coal or shale. FTIR characterization of coal samples before and after NO treatment revealed the formation of amide groups in coal after NO exposure [56]. It was found that the newly formed amine could act as a Lewis base to donate electrons to acidic CO_2_ and resultantly intensify shale attraction toward CO_2_, thus effectively adsorbing CO_2_. That is, the affinity to CO_2_ increased as the amide content of the matrix increased [57].

N_2_ preferentially exists as free gas in coal and shale pore spaces due to its low solubility and slow adsorption rate, as evidenced by its short breakthrough time [58]. Its presence forms a diffusion medium, promoting a more uniform distribution of CO_2_-IWG in pores. This helps alleviate the significant expansion of the coal and shale matrix caused by CO_2_ and NO adsorption, which can reduce reservoir permeability by at least an order of magnitude [59]. The high diffusivity of N_2_ also improves the overall dynamic balance of the gas mixture [60].

## 4. Conclusions

In this study, we investigated the effects of CO_2_-rich IWG treatment on the pore structure of a coal-bearing rock series, including the SSA, PV, and PSD. To ensure a comprehensive analysis across a range of maturities from low to high ranks, lignite, bituminous, and anthracite coal samples and a shale sample were collected from four mines and treated with CO_2_-rich IWG for 15 days. The changes in the pore structure and adsorption isotherms of the samples caused by the treatment were analyzed. In addition, the relationship between micropores and mesopores and adsorption capacity, as well as between macropores and pore connectivity, were investigated in detail. This study shows that CO_2_-rich IWG can effectively change the pore structures of coal and shale, improve their adsorption performance, and have a positive impact on the potential for long-term gas storage. These findings provide valuable insights into the mechanisms of CO_2_-rich IWG treatment and offer a theoretical foundation for its application. Future optimization of treatment conditions could focus on improving the efficiency of pore structure modification. The main conclusions of this study are summarized below.

(1) For the coal samples, the PV and SSA of micropores increased with increasing coal rank, while those of mesopores and macropores exhibited inverted V-shaped trends. For the shale, the PV and SSA of micropores and macropores differed by several orders of magnitude compared to the coal samples.
(2) The CO_2_-rich IWG treatment resulted in improvement in the PV and SSA of micropores in the samples, most notably in the shale. The underlying reasons for this are similar, that is, the treatment dissolved blockages and thus increased the size of the major pores. The difference in the PSDs of the micropores in the coals and shale is that the coals had two peaks in their curves, while the shale had three peaks. The coals and shale shared the characteristic that the peaks were distributed within the 0.4–0.7 nm pore diameter range.
(3) The CO_2_-rich IWG treatment promoted the development of mesopores in the shale, but it had a negative impact on the bituminous coal, decreasing the SSA and PV of mesopores, leading to larger main pore diameter. The lignite and anthracite responded similarly to the CO_2_-rich IWG treatment, with increased PVs and smaller main pore diameters, which partially offset the adverse effect of the decreases in their SSAs.
(4) The CO_2_-rich IWG treatment increased the PVs of the macropores in all of the samples, except for the lignite, and the anthracite experienced the most pronounced improvement. The SSAs of macropores in the coal samples decreased, whereas those of the shale increased. The pore structures of all of the samples became more complex, and only the main pore diameter of the bituminous coal became larger.
(5) The excess adsorption capacities of the samples underwent significant enhancement, and the shale experienced the largest improvement. The PVs and SSAs of micropores and mesopores were positively correlated with their adsorption capacity. The SSA of micropores accounted for the dominant proportion. Changes in adsorption capacity need to be analyzed by considering multiple parameters together. The CO_2_-rich IWG treatment increased the volume of effectively interconnected pores in the samples, except for the lignite.
(6) The component gases of CO_2_-rich IWG have different roles in the treatment process, and the mechanisms are as follows: CO_2_ leads to dissolution of minerals, NO changes matrix structures, and N_2_ maintains the balance of gas diffusion. The results of this study provide a theoretical basis for the application of direct sequestration of CO_2_-rich IWG.

## Figures and Tables

**Table 1:** Properties of the coal samples and shale sample.

Samples	*R*_o_ (%)	Proximate (wt. %)				Ultimate (wt.%)			
		*M*_ad_	*A*_ad_	*V*_ad_	*FC*_ad_	*C*_daf_	*O*_daf_	*N*_daf_	*H*_daf_
1 Lignite	0.36	6.06	10.07	40.41	45.22	58.67	14.87	0.90	4.80
2 Bituminous coal	0.61	5.52	3.78	33.90	56.80	72.68	9.90	0.86	4.94
3 Anthracite	2.98	1.28	9.02	10.28	79.42	82.85	8.65	0.56	2.92
4 Shale	2.68	0.06	55.96	16.02	27.96	13.81	15.30	0.14	0.68

*Note: R*_o_—mean random vitrinite reflectance; wt. %—weight percent; *M*_ad_—moisture; *A*_ad_—ash yield; *V*_ad_—volatile matter; *FC*_ad_—fixed carbon; *C*_daf_—carbon; *H*_daf_—hydrogen; *O*_daf_—oxygen; *N*_daf_—nitrogen; ad denotes air-drying basis; daf denotes dry ash-free basis.

**Table 2:** Langmuir fitting parameters for the samples.

Samples		Untreated			Treated	
	*R* ^2^	*V_L_*	*P_L_*	*R* ^2^	*V_L_*	*P_L_*
1 Lignite	0.9945	24.5046	1.4016	0.9696	27.2727	1.5382
2 Bituminous coal	0.9928	32.3365	1.6213	0.9764	30.8333	1.2803
3 Anthracite	0.9974	34.2331	0.7915	0.9882	34.7564	0.9651
4 Shale	0.9813	1.0963	1.7683	0.9721	2.4479	1.5794

*Note: V_L_*—Langmuir volume (cm^3^/g); *P_L_*—Langmuir pressure (MPa); *R*^2^—correlation coefficient, dimensionless.

**Table 3:** Volumes of effectively interconnected pores and non-effectively interconnected pores before and after CO_2_-rich IWG treatment.

Samples	Untreated		Treated		Δ*V_Inter_*	Δ*V_Clo_*
	*V_Inter_*	*V_Clo_*	*V_Inter_*	*V_Clo_*		
1 Lignite	0.0452	0.0556	0.0425	0.0507	−5.97%	−8.81%
2 Bituminous coal	0.0484	0.0609	0.0550	0.0854	13.64%	40.22%
3 Anthracite	0.0228	0.0294	0.0215	0.0815	−5.70%	177.21%
4 Shale	0.0020	0.0011	0.0021	0.0015	5.00%	36.36%

*Note:* $V_{Inter}$ is the volume of effectively interconnected pores, cm^3^/g; $V_{Clo}$ is the volume of non-effectively interconnected pores, cm^3^/g; and Δ*V_Inter_* and Δ*V_Clo_* are the rates of change of $V_{Inter}$ and $V_{Clo}$ after CO_2_-rich IWG treatment.

## Data Availability

All data underlying the results are available as part of the article, and no additional source data are required.
